# 

**Published:** 1911-10

**Authors:** 


### CONTENTS.

ORIGINAL COMMUNICATIONS

Foods and Their Calorific Value. By G. Bachmann, M. D. .................. 345

Typhoid Fever. Dr. Thomas Northen, Ashland, Alabama ...................... 352

Carcinoma of the Uterus and Some of Its Problems. W. F. Shallenberger, M. D. Atlanta, Ga. .................. 361

The Wertheim-Watkins Operation for Cystocele. By R. R. Kime, M. D., Atlanta, Ga. .................. 368

The Sterilization of Degenerate Criminals. By Tom A. Williams, M. D., C. M., Washington, D. C. .................. 371

Hay Fever. By H. S. Persons, M. D. .................. 373

EDITORIALS .................. 383

NEWS AND NOTES .................. 384

BOOK REVIEWS .................. 396

